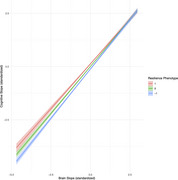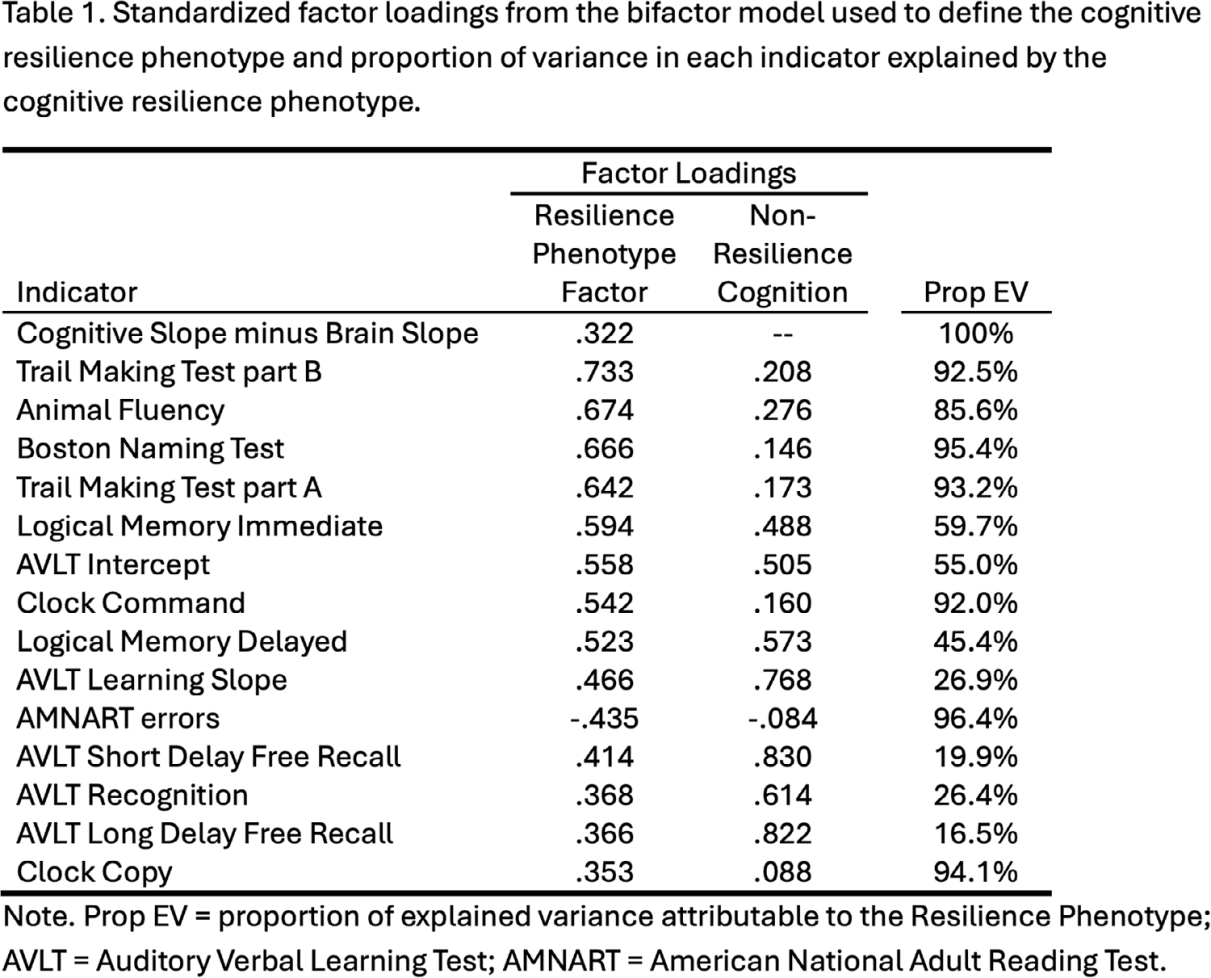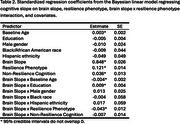# A cognitive ability profile can provide a valid phenotype for future cognitive resilience to brain aging

**DOI:** 10.1002/alz70857_107458

**Published:** 2025-12-26

**Authors:** Brandon E Gavett, Evan Fletcher, Sarah Tomaszewski Farias, Keith F. Widaman, Dan M. Mungas

**Affiliations:** ^1^ University of California Davis, Sacramento, CA, USA; ^2^ Department of Neurology, University of California, Davis, Davis, CA, USA; ^3^ University of California, Davis School of Medicine, Sacramento, CA, USA; ^4^ University of California at Riverside, Riverside, CA, USA

## Abstract

**Background:**

Individual differences have been observed in the association between neurodegenerative brain changes and rates of cognitive decline. People who experience less rapid decline than expected based on neurodegeneration are described as being cognitively resilient to brain aging. We hypothesized that patterns of cognitive strengths and weaknesses (certain cognitive profiles) can provide a valid phenotype of prospective resilience.

**Method:**

Participants (*N* = 2432) were from the Alzheimer's Disease Neuroimaging Initiative (ADNI) study (1/2/3/GO). There were three analytic phases: 1) generate an estimate of prospective resilience; 2) use that resilience estimate to develop a baseline cognitive resilience phenotype; 3) validate the phenotype longitudinally. In the first phase, prospective resilience was defined as the discrepancy between rates of future cognitive and brain change (cognitive slope minus brain slope). In the second phase, a bifactor confirmatory factor analysis model defined the cognitive resilience phenotype as a latent variable representing the variance that the observed cognitive test scores shared with the prospective resilience estimate from phase 1. In the third phase, we used regression in a held‐out sample to test the hypothesized interaction between the resilience phenotype and brain volume change when predicting longitudinal cognitive decline (i.e., does the phenotype modify the association between brain atrophy and rate of cognitive decline?).

**Result:**

The cognitive performance indicators that most strongly contributed to the resilience phenotype were Trail Making Test B (loading = .73), animal fluency (loading = .67), and Boston Naming Test (loading = .67; Table 1). The cognitive resilience phenotype moderated the regression of cognitive slope on brain slope in the held‐out sample (B=‐0.043, SE=0.016; Table 2, Figure 1).

**Conclusion:**

A cross‐sectional estimate of prospective cognitive resilience against brain atrophy can be constructed from the ADNI neuropsychological test battery. This phenotype, which draws heavily upon measures of processing speed/executive functioning, category fluency, verbal naming, and immediate recall, can predict the extent to which future brain atrophy will impact rates of cognitive decline. Examining cognitive phenotypes associated with more resilient cognitive aging may help to understand neural systems that underpin resilience, potentially leading to resilience‐promoting interventions and new opportunities to efficiently monitor changes in resilience over time.